# Comorbidity profiles among sputum-positive tuberculosis patients in Cameroon

**DOI:** 10.3389/ftubr.2024.1433856

**Published:** 2024-10-02

**Authors:** Chefor Magha, Lucy Cho Nchang, Michael Weldeslassie, Desmond Akumtoh Nkimbeng, Nancielle Mbiatong Tchatat, Henry Dilonga Meriki, Kebede Deribe, Frank Noel Nietcho, Juluis Visnel Foyet, Fanny Fri Fombad, Tatiana Djikeussi Katcho, Jerome Fru Cho, Eyoab Iyasu Gebremeskel, Simon J. Waddell, Kidist Bobosha, Melanie J. Newport, Achim Hoerauf, Manuel Ritter, Samuel Wanji

**Affiliations:** 1 Parasites and Vector Research Unit (PAVBRU), Department of Microbiology and Parasitology, University of Buea, Buea, Cameroon; 2 Research Foundation for Tropical Diseases and the Environment (REFOTDE), Buea, Cameroon; 3 Department of Biology, Mai Nefhi College of Science, Mai Nefhi, Eritrea; 4 Children’s Investment Fund Foundation, London, United Kingdom; 5 Department of Global Health and Infection, Brighton and Sussex Medical School, University of Sussex, Brighton, United Kingdom; 6 Armauer Hansen Research Institute (AHRI), Addis Ababa, Ethiopia; 7 Institute for Medical Microbiology, Immunology and Parasitology (IMMIP), University Hospital Bonn (UKB), Bonn, Germany; 8 German Center for Infection Research (DZIF), Partner Site Bonn-Cologne, Bonn, Germany; 9 German-West African Centre for Global Health and Pandemic Prevention (G-WAC), Partner Site Bonn, Bonn, Germany

**Keywords:** *Mycobacterium tuberculosis*, comorbidities, non-communicable diseases, tuberculosis, diabetes, cardiovascular disease, kidney disease

## Abstract

**Introduction:**

Comorbid non-communicable diseases (NCDs) like diabetes, cardiovascular diseases (CVD), kidney diseases, and hypertension, could have implications for tuberculosis (TB) treatment management and increase the disease burden amongst active TB patients.

**Methods:**

This cross-sectional study aimed at profiling comorbidities amongst sputum-positive TB patients in the South West and Littoral regions of Cameroon and was relevant for improving disease management and public health interventions. Diabetes was defined by elevated blood glucose, body mass index (underweight: <18.5 kg/m^2^, normal: 18.5–<25.0 kg/m^2^, overweight: 25–<30 kg/m^2^ and obese: ≥30.0 kg/m^2^) and hypertension by elevated blood pressure levels (i.e., systolic ≥130 mmHg or diastolic ≥80 mmHg). Socio-demographic and clinical data were collected using case report forms. Descriptive analysis was performed, bivariate logistic regression analysis was computed with at least one comorbidity as the dependent variable (global model) and a multivariable logistic regression analysis was done to provide adjusted odds ratios (final model). The covariate with the highest *p*-value was removed until *p* < 0.25 cut-off, using R software version 4.3.1. *p*-value <0.05 at 95% confidence interval was considered statistically significant.

**Results:**

Five hundred and forty-nine sputum-positive microscopically confirmed active TB patients were enrolled into this study. Two-thirds (65.8%) of the total patients were male. Overall, 56 sputum-positive TB patients had at least one non-communicable disease, thus a prevalence of 10.2% (95% CI = 7.9–13.0). The most frequently recorded NCD was diabetes 4.4% (95% CI = 3.1–6.7) followed by kidney disease 2% (95% CI = 1.1–3.6), hypertension 0.9% (95% CI = 0.4–2.2), and CVD 0.91% (95% CI = 0.4–2.2). Three TB patients (0.6%) had all four comorbidities examined. Age group (*p* < 0.001), and level of education (*p* = 0.049) were factors significantly associated with having at least one comorbidity.

**Discussion:**

Our findings showed that diabetes was significantly the most prevalent comorbid NCD amongst sputum-positive TB patients (*p* < 0.001). HIV status, occupation, body mass index (BMI), and alcohol intake were not significantly associated with having at least one comorbidity. Implementing public health intervention programmes such as systematic screening of TB patients for NCDs especially diabetes is highly recommended for better control of these diseases.

## Introduction

Tuberculosis (TB) is caused by the bacillus *Mycobacterium tuberculosis* and is the world’s second leading cause of death from a single infectious agent after COVID-19 (1). TB is among the oldest endemic diseases affecting humans and still poses a significant global public health problem (2, 3). Transmission of tuberculosis usually happens when an individual with active pulmonary tuberculosis coughs, sneezes, speaks or sings, releasing aerosolized droplets containing infectious tubercle bacilli that are inhaled by an uninfected person (4, 5). The global number of people newly diagnosed with TB in 2022 was 7.5 million, this represents the highest number since the World Health Organization (WHO) began global TB monitoring in 1995 (1). On the other hand, non-communicable diseases (NCDs) have long-term health consequences and are non-transmissible. They are caused by a combination of genetic, physiological, environmental, and behavioral factors and disproportionately affect people in low and middle-income countries, where more than three-quarters of global NCD deaths (31.4 million) occur (6). NCDs and tuberculosis are among the top 10 causes of and share common risk factors like smoking, alcohol abuse, physical inactivity and inadequate vegetable and fruit consumption (6–8). NCDs like heart disease, hypertension, chronic obstructive pulmonary diseases (COPD), cancer, diabetes mellitus, alcohol use disorders and smoking-related conditions are most common in TB patients (9, 10). Pulmonary tuberculosis is increasingly diagnosed among individuals with common mental disorders and alcohol and substance abuse, contributing substantially to the rise of multi-drug-resistant tuberculosis (MDR-TB) in many countries as a result of non-compliance (11–14). Meta-analysis of studies from all continents calculated a two–four fold increased risk for TB in patients with diabetes mellitus, with the concurrence of TB and diabetes mellitus being more prevalent than TB and HIV (15). People with diabetes also show impaired sputum conversion and cure rates on tuberculosis treatment, with increased risk of death and relapse (16, 17). Furthermore, the estimated prevalence of hypertension among patients with TB ranges between 0.7 and 38.3% (18) and TB patients have also been reported to significantly have higher risks of developing chronic kidney disease (CKD) (19). Although, the mechanism of how NCDs might negatively affect the dynamics of the TB epidemic is still under research (20). It is mandatory to consider NCDs as an important factor with regards to TB management by control programmes. Indeed, it has been shown that the absence of reports on NCD co- and multi-morbidity amongst TB patients may harm the success of TB control programmes (14, 21). Currently, there are limited studies that have described the prevalence of comorbidities amongst TB patients in Cameroon. Additionally, TB treatment is lengthy and other chronic comorbid diseases could have significant consequences on disease burden as well as implications for treatment management. This study therefore aimed at profiling non-communicable disease comorbidities amongst sputum-positive TB patients diagnosed by microscopy in the South West and Littoral regions of Cameroon.

## Methods

### Study site

**Figure 1:**
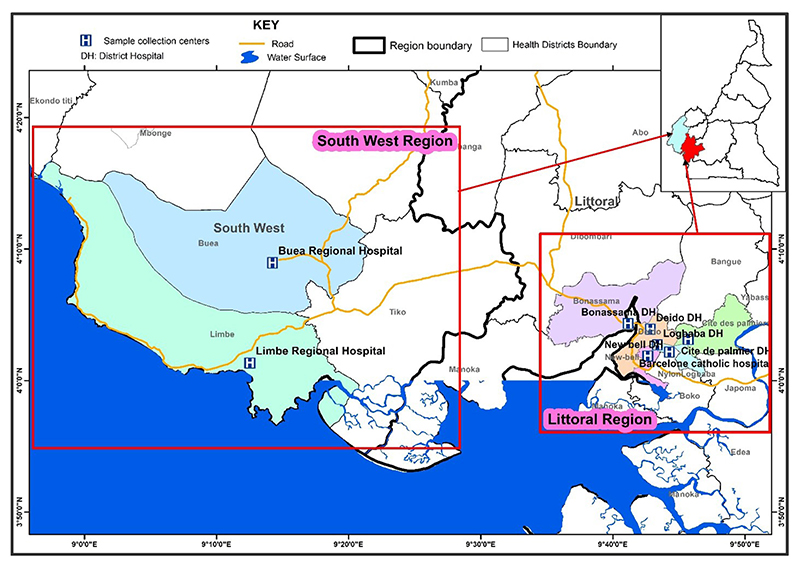
Map showing the eight TB diagnostic and treatment centers selected in the South West and Littoral regions of Cameroon.

This study was carried out in the Littoral and South West regions of Cameroon in the cities of Douala, Limbe and Buea. Douala is the economic capital of Cameroon. The city and its surrounding area have an estimated population of 3.9 million (22), with twenty TB treatment and diagnostic centers. Limbe and Buea are towns located in Fako Division-South West region of Cameroon, with seven TB treatment and diagnostic centers. As of 2015, the population of the South West region was 1,534,232 (23). Eight out of 27 TB diagnostics and treatment centers were randomly selected for the study: two from the South West and six from the Littoral regions (Figure 1).

### Study design

This was a cross-sectional study carried out among newly diagnosed sputum-positive TB cases aged 15 years and above from February 2020 to February 2022. Demographic information, vital signs, previous TB infection history, medical history of NCDs [diabetes, hypertension, cardiovascular diseases (CVD), and kidney disease], as well as other infections, alcohol consumption and cigarette smoking were prospectively collected by trained healthcare personnel from individual hospital records and verbal self-reports using structured questionnaires that partially followed WHO guidelines for NCDs self-reporting (dietary habits and physical activity were not recorded) before the onset of TB treatment.

### TB and NCD diagnosis

TB diagnosis and treatment in Cameroon is the responsibility of the National TB Control Programme under the Ministry of Public Health. Individuals with symptoms suggestive of TB (such as cough, fever, night sweats and weight loss) were sent to tuberculosis diagnostic and treatment centers for laboratory diagnosis, including sputum smear microscopy and Xpert MTB/RIF assay or the TB-LAM assay according to national guidelines. Only TB patients tested sputum-positive by microscopy were enrolled into this study. CRFs were used to collect sociodemographic information. Vital signs, height, and weight were measured and recorded on the CRFs. NCD information was obtained from self-reports of the patients based on current medical history. In that regard, medical hospital records information about diabetes, hypertension, cardiovascular diseases (CVD), kidney disease, alcohol consumption and cigarette smoking were obtained. Diabetes was defined by increased blood glucose levels (fasting glucose: >126 mg/dl, random glucose: >200 mg/dl), obesity by Body Mass Index (BMI; underweight: <18.5 kg/m^2^, normal: 18.5–<25.0 kg/m^2^, overweight: 25–<30 kg/m^2^ and obese: ≥30.0 kg/m^2^ (24), hypertension by increased blood pressure (systolic ≥130 mmHg or diastolic ≥80 mmHg), kidney disease (albumin-to-creatinine ratio >30 mg/g in two of three spot urine specimens) and CVD (records of heart and blood vessel disorders). Participants of the study and also individuals who declined the enrolment started their anti-TB treatment based on national guidelines.

### Study population and selection criteria

For enrolment into the study, the participants or legal guards had to provide informed consent. Moreover, participants have to be above 15 years old, had a sputum-positive smear and did not start treatment for TB. Pregnant women and individuals who were disoriented, in severe distress, severely anemic, unconscious or mentally incapacitated individuals, or those with bleeding tendencies were not enrolled in the study but received TB treatment according to the national guidelines.

### Sample size calculation

The sample size was calculated based on the formula for sample size calculation for cross-sectional studies as follows

$$n=\frac{\left[z_{2}^{2}z_{2}p\;(1-p)\right]}{m_{2}}=\frac{\left[1.96_{2}^{2}\times 0.5\;(1-0.5)\right]}{\left[0.05\right]}=384$$

Where *n* is the sample size, *z* = 1.96 is the critical value of the confidence interval for a standard normal distribution (for 95% confidence intervals). *P* = 0.5 is an estimated population proportion that produces the largest sample size (for a given value of m). The value of 50% was used because, at the time of this study, there was no previous study on comorbidity prevalence rates in bacteriologically confirmed TB patients in Cameroon, and *m* = 0.05 is the required precision. The minimum estimated sample size was 384 cases. However, this study enrolled 549 sputum-positive TB patients.

### Data processing

Data were entered into Microsoft Excel 2016 and checked for completeness. R software version 4.3.1 was used for simple and multiple binary logistic regression analysis. Descriptive analysis was performed, and frequency distribution of comorbidity combinations included only sputum-positive TB patients having at least one comorbidity. The outcome variable was the presence of at least one NCD (diabetes, hypertension, CVD, or kidney disease) amongst sputum-positive TB patients. The predictor variables assessed as associated risk factors for comorbidity were sex, age group, occupation, education level, health facility, cigarette smoking, BMI, alcohol intake, and HIV status. Multiple binary logistic regression analysis including all the covariates was performed for the global model. The final model was obtained by a step-wise backward deletion of covariates from the global model. For each step of deletion, the covariate with the highest *p*-value was removed until *p* < 0.25 cutoff. Crude and adjusted odds ratios, as well as their 95% confidence intervals were deduced from those models. A statistically significant difference was set at *p* < 0.05. Results were presented using frequencies (percentages) in tables and charts.

### Ethics

The study was cleared by the National Ethics Committee for Human Research, Yaoundé (CNERSH) No. 2019/03/1154/CE/CNERSH/SP. Written informed consent was sought and obtained for all participants before enrolment.

## Results

### General characteristics of the study population

Five hundred and forty-nine sputum-positive TB patients were enrolled into this study. Two-thirds (65.8%) of the total patients were male. The distribution of participants according to their sex, age groups, occupation, education level, health facility, cigarette smoking, alcohol intake, BMI and HIV status are shown in Table 1. The highest number of sputum-positive TB patients came from Barcelone Catholic Hospital (135/459), and 87.4% of all sputum-positive TB patients were HIV-negative. Most of these patients (75.6%) did not smoke cigarettes, (63.9%) had normal BMI, and the majority of them were artisans and businessmen/women (18.6 and 15.9%, respectively).

### Frequency distribution of comorbidity combinations in sputum-positive TB patients having at least one comorbidity

**Figure 2:**
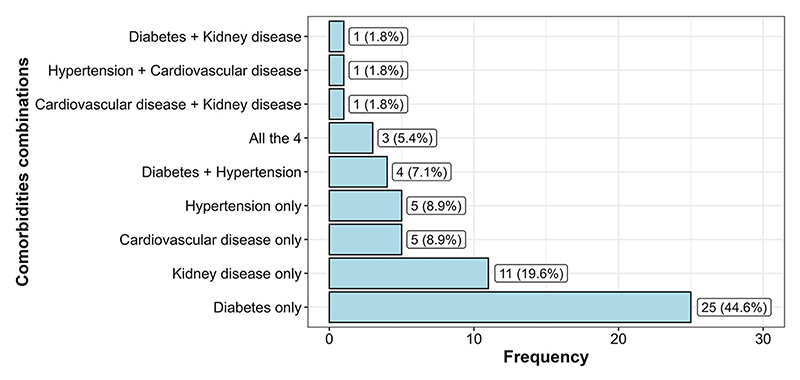
Frequency distribution of comorbidity combinations in sputum-positive TB patients having at least one comorbidity.

Overall, 56 sputum-positive TB patients had at least one non-communicable disease, thus a prevalence of 10.2% (95% CI = 7.9–13.0). The most frequently recorded NCD was diabetes with 4.4% (95% CI = 3.1–6.7); *p* <0.001, followed by kidney disease with 2% (95% CI = 1.1–3.6), hypertension with 0.91% (95% CI = 0.4–2.2), and CVD with 0.91% (95% CI = 0.4–2.2). Three sputum-positive TB patients (0.6%) had all four comorbidities examined as shown in Figure 2. Diabetes and hypertension multi-morbidity was recorded in four TB patients. CVD and kidney disease multi-morbidity were also found in one TB patient. This was also the case for diabetes and kidney disease multi-morbidity as well as hypertension and CVD multi-morbidity.

### Socio-demographic, clinical, and behavioral factors associated with tuberculosis and comorbidities

Based on the global model, age group (*p* < 0.001), and level of education (*p* = 0.049) were two factors associated with having at least one comorbidity. From the final model, sputum-positive TB patients who were 45 years and above had significantly higher odds of having at least one comorbidity (*p* < 0.001) when compared to patients between 15 and 24 years of age. TB patients with primary school education had significantly higher odds of having at least one comorbidity when compared to TB patients with high school education (AOR, 2.0; 95% CI: 1.0–4.1). TB patients who smoke cigarettes were less likely to be found with at least one comorbidity as compared to sputum-positive TB patients who did not smoke cigarettes (AOR, 0.4; 95% CI: 0.2–0.9, *p* = 0.040). Sex, occupation, health facility, alcohol intake, BMI, and HIV status were not seen as factors associated with having at least one comorbidity in sputum-positive TB patients as shown in Table 2.

## Discussion

Tuberculosis, as well as a wide range of comorbid NCDs are prevalent and overlapping in most developing countries. There are already some studies reporting comorbidities among TB cases in Sub-Saharan Africa, particularly diabetes and hypertension. However, data sets about distinct NCDs, especially cardiovascular and kidney diseases are limited and almost no reports have been published from rural areas as shown here with study sites in the South West and Littoral regions of Cameroon. Thus, we aim to fill this gap of knowledge and assessed comorbid diabetes, hypertension, kidney disease, and cardiovascular diseases among sputum-positive TB patients before their enrolment into the direct observed treatment (DOT) in tuberculosis diagnostic and treatment centers. Our study found that 10.2% were comorbid with at least one NCD assessed. This figure is higher than 7.22% reported in Ethiopia (25) and lower than 26.9% in South Africa (26). These variations might be due to differences in NCD prevalence in different communities. This study found a higher proportion of males suffering from TB than females. The higher share of TB cases among men is consistent with evidence from global reports showing similar patterns (1, 27, 28), as well as surveys conducted in low- and middle-income countries (29).

The most frequently recorded comorbid NCD amongst TB patients in this study was diabetes with a prevalence of 4.6%. These findings are higher than reports of 1.6% in Coutonou-Benin (30), and lower than the 5.7% prevalence in Nigeria (31), 6.7% in Tanzania and Kenya (32, 33), 8.5% in Uganda (34), 9.5% in Cameroon (35), and 16% in Ethiopia (36). Reasons for these differences could be variations in sample size and lifestyle of TB patients in the study sites. Kidney disease was the second most frequently recorded comorbid NCD amongst sputum-positive TB patients in this study with a prevalence of 2%. These results are lower than the 5.24% prevalence of chronic kidney disease from a study carried out amongst pulmonary and extra-pulmonary TB patients in Cameroon (37). Hypertension was the third most frequently recorded comorbid NCD amongst TB patients in this study with a prevalence of 0.9%. These findings are higher than reports of 0.7% in Brazil (38) and lower than reports of 19% in Angola (39). The very low prevalence of comorbid hypertension in TB patients in Brazil could be because data entry on comorbidity in that study was optional. This study recorded a 0.91% prevalence of cardiovascular diseases. These findings are lower than reports of 11% pooled prevalence from a systematic review of CVD among TB patients (40).

Sputum-positive TB patients aged 45 years and above in this study were more likely at risk for at least one comorbidity when compared to the 15–24 years age group. Several studies have reported that older age increases the risk of TB and diabetes comorbidity (41, 42). These findings are in line with other studies where older age was also associated with hypertension in tuberculosis patients (39, 43). Generally, other underlying factors likely influence the blood pressure dynamics in active TB patients. In addition, a study carried out in Taiwan also showed that the incidence of chronic kidney disease was higher in TB patients with age over 50 years (19). These comorbid conditions among active TB patients could have implications for treatment management. Our findings showed that sputum-positive TB patients with primary school education had significantly higher odds of having at least one comorbidity when compared to TB patients with high school education. Contrary to these findings, another study indicated that having education beyond primary schooling was an associated factor to TB-diabetes comorbidity (44).

We found that active TB patients who smoke cigarettes had lower odds for at least one comorbidity. Contrary to these findings, smoking has been reported as risk factor for NCD (45) and diabetes in TB patients (46). BMI, HIV status and alcohol intake were not seen as factors associated to having at least one comorbidity in sputum-positive TB patients. Contrary to these findings, a study carried out in China reported overweight and obesity to be significantly associated with diabetes in pulmonary TB patients (47), similar to a study carried out in Angola where BMI was associated with hypertension (39). Furthermore, other studies have reported HIV status (48, 49) and alcohol intake as factors associated with comorbid hypertension (43) in TB and diabetes patients (50). The reasons for these differences in associated risk factors to comorbidities in active TB patients in various studies could range from behavioral and societal factors amongst individuals in study areas as well as differences in study designs. TB treatment success is very crucial for TB management strategies, especially in low- and middle-income countries, as treatment failure increases the incidence of active TB as well as multidrug resistance. Comorbid NCDs generally have implications for the management of TB disease. One important factor contributing to TB treatment failure is comorbidity which may eventually lead to death (51, 52). Comorbid diabetes in TB patients leads to a number of negative TB treatment consequences including increased drug resistance in TB patients, TB treatment prolongation and failure (53–55). The “WHO Collaborative Framework of Care and Control of Tuberculosis and Diabetes” (56) recommended for healthcare professionals to ensure effective management of both diseases, should be diligently followed in TB diagnostic and treatment centers nation-wide. Administering the appropriate treatment for TB patients with chronic kidney disease is of great importance in reducing mortality, as inappropriate dosage of anti-TB drugs can result in drug-related side effects or unsuccessful treatment (57). Hypertension has been associated with increased mortality in patients with tuberculosis (58). Tuberculosis may also contribute to the pathogenesis of CVD (59). Given that undiagnosed comorbid NCDs in tuberculosis patients might negatively influence the success of TB control programmes (21), developing an integrated platform for systematic screening of active TB patients for comorbid NCDs is a great strategy for targeting comorbidities in TB patients and hence enhance disease management as well as treatment success rates.

### Limitations

Although we confirmed the diagnosis of tuberculosis, as in many observational studies, information on NCDs was obtained from individual hospital record books and verbal self-reports. It is possible that several cases were missed because of no concurrent diagnosis of comorbid NCDs, and the unwillingness of patients to declare their health status. Information based on self-reports could not be verified for accuracy. In addition, other risk factors such as dietary habits, and physical activity were omitted from the data collection and information about concomitant medication against NCDs have been not collected. Therefore, these findings should be interpreted with caution. Another limitation of the study results is that only sputum-positive TB patients were enrolled into the study. It is known that coinfections like HIV can reduce the positivity of sputum smears and thus it might be that some TB cases were not detected. However, Xpert MTB/RIF or TB-LAMP assay was not available in most of the TB centers, we were forced to rely on the microscopical diagnosis. Altogether, the limitations of the study especially in regards to the undetected TB and NCD cases might even lead to an underestimation of the overlapping prevalences of TB and NCDs, highlighting the need for improved TB and NCD diagnosis.

## Conclusions

Sputum-positive TB patients aged 45 years and above in this study had higher odds of having at least one comorbidity. Diabetes was the most frequently recorded comorbid NCD amongst sputum-positive TB patients. HIV status, occupation, BMI, and alcohol intake were not significantly associated with having at least one comorbidity. Concurrent diagnosis of NCDs amongst TB patients is highly recommended since it will shed more light on these overlapping disease conditions and might support disease management and control, ultimately reducing the disease burden and contributing to the WHO End TB Strategy.

## Figures and Tables

**Table 1:** General characteristics of the study population.

Variables	Category	Frequency	Percent
Sex	Female	188	34.2
	Male	361	65.8
Age range	15–24	106	19.3
	25–34	196	35.7
	35–44	125	22.8
	45–54	58	10.6
	55–64	47	8.6
	≥65	17	3.1
Occupation	Artisan	102	18.6
	Business	87	15.9
	Salaried	78	14.2
	Student	66	12.0
	Transporter	54	9.8
	Unemployed	46	8.4
	Farmer	40	7.3
	Others	76	13.8
Education level	High school	209	38.1
	Primary school	233	42.4
	Diploma and above	74	13.5
	None	33	6.0
Health facility	Barcelone Catholic Hospital	135	24.6
	Cite Des Palmiers District Hospital	118	21.5
	Limbe Regional Hospital	79	14.4
	Deido District Hospital	60	10.9
	Newbell District Hospital	51	9.3
	Logbaba District Hospital	47	8.6
	Buea Regional Hospital	45	8.2
	Bonassama District Hospital	14	2.6
Cigarette smoking	No	415	75.6
	Yes	134	24.4
Alcohol intake	No	228	41.5
	Yes	321	58.5
BMI	Underweight	149	27.1
	Normal	351	63.9
	Overweight	40	7.3
	Obesity	9	1.6
HIV status	Negative	480	87.4
	Positive	69	12.6

**Table 2:** Determinants of tuberculosis and at least one comorbidity.

Variables	Total	Prevalence	Global model		Final model	
			OR (95% CI)	*p*-value	AOR (95% CI)	*p*-value
**Sex**				0.261		
Female	188	23 (12.2)	Reference	–		
Male	361	33 (9.1)	0.7 (0.3–1.6)	0.434		
**Age range**				<**0.001**		<**0.001**
15–24	106	5 (4.7)	Reference	–	Reference	–
25–34	196	8 (4.1)	1.2 (0.3–4.9)	0.843	0.88 (0.3–3.0)	0.823
35–44	125	6 (4.8)	1.6 (0.3–8.1)	0.674	1.12 (0.3–4.1)	0.860
45–54	58	14 (24.1)	11.2 (2.7–55.6)	**0.002**	8.53 (2.9–29.1)	<**0.001**
55–64	47	17 (36.2)	19.1 (4.7–96.6)	<**0.001**	14.7 (5.0–50.2)	<**0.001**
≥65	17	6 (35.3)	23.5 (4.2–151)	<**0.001**	14.0 (3.4–61.3)	<**0.001**
**Occupation**				0.794		
Artisan	102	7 (6.9)	Reference	–		
Business	87	10 (11.5)	2.0 (0.6–7.0)	0.347		
Farmer	40	6 (15.0)	0.9 (0.2–3.9)	0.833		
Salaried	78	7 (9.0)	1.4 (0.4–5.4)	0.633		
Student	66	4 (6.1)	2.5 (0.4–14.8)	0.321		
Transporter	54	6 (11.1)	2.3 (0.6–8.7)	0.226		
Unemployed	46	5 (10.9)	0.7 (0.2–2.9)	0.620		
Others	76	11 (14.5)	2.4 (0.6–7.4)	0.209		
**Education level**				0.070		**0.040**
High school	209	16 (7.7)	Reference	–	Reference	–
Primary school	233	33 (14.2)	1.9 (0.9–4.0)	0.112	2.0 (1.0–4.1)	**0.049**
Diploma and above	74	6 (8.1)	1.5 (0.4–5.2)	0.516	2.0 (0.6–5.8)	0.212
None	33	1(3.0)	0.3 (0.0–2.2)	0.319	0.4 (0.0–2.5)	0.433
**Health facility**				0.373		
Barcelone Catholic Hospital	135	13 (9.6)	Reference	–		
Bonassama District Hospital	14	2 (14.3)	0.6 (0.1–4.8)	0.713		
Buea Regional Hospital	45	3 (6.7)	0.9 (0.2–3.8)	0.905		
Cite des Palmiers District Hospital	118	16 (13.6)	1.7 (0.6–4.7)	0.298		
Deido District Hospital	60	4 (6.7)	0.9 (0.2–3.3)	0.914		
Limbe Regional Hospital	79	13 (16.5)	2.1 (0.7–6.5)	0.173		
Logbaba District Hospital	47	4 (8.5)	0.9 (0.2–3.2)	0.824		
Newbell District Hospital	51	1 (2.0)	0.3 (0.0–1.9)	0.273		
**Cigarette smoking**				**0.030**		**0.020**
No	415	48 (11.6)	Reference	–	Reference	–
Yes	134	8 (6.0)	0.4 (0.1–0.9)	0.054.	0.4 (0.2–0.9)	**0.040**
**Alcohol intake**				0.723		
No	228	22 (9.6)	Reference	–		
Yes	321	34 (10.6)	1.2(0.6–2.5)	0.683		
**BMI**				0.315		0.380
Underweight	149	14 (9.4)	Reference	–	Reference	–
Normal	351	28 (8.0)	0.7 (0.3–1.5)	0.286	0.7 (0.35–1.6)	0.421
Overweight	40	12 (30.0)	1.7 (0.5–5.5)	0.408	1.7 (0.56–4.8)	0.411
Obesity	9	2 (22.2)	0.6 (0.1–4.0)	0.629	0.7 (0.1–3.9)	0.730
**HIV status**				0.997		
Negative	480	47 (9.8)	Reference	–		
Positive	69	9 (13.0)	1 (0.4–2.5)	0.997		

Significant values are in bold.

## Data Availability

The original contributions presented in the study are included in the article/supplementary material, further inquiries can be directed to the corresponding authors.

## Abbreviations

TB: tuberculosis
HIV: Human Immunodeficiency Virus
NCDs: non-communicable diseases
OR: odds ratio
AOR: adjusted odds ratio
WHO: World Health Organization
COPD: chronic obstructive pulmonary diseases
DM: diabetes mellitus
MDR-TB: multi-drug-resistant tuberculosis
CKD: chronic kidney disease
CVD: cardiovascular disease
ZN: Ziehl-Nelson
AFB: acid-fast bacilli
LJ: Lowenstein-Jensen
BMI: body mass index
PBS: phosphate buffer saline
